# Beneficial Effect of Polydeoxyribonucleotide in the Treatment of Actinic Cheilitis: A Case Report

**DOI:** 10.7759/cureus.98756

**Published:** 2025-12-08

**Authors:** Pilar Jara

**Affiliations:** 1 Aesthetic Medicine, Dr. Pilar Jara Academy, Santiago, CHL

**Keywords:** actinic cheilitis, clinical case report, polydeoxyribonucleotide, primary prevention of cancer, regenerative medicine therapies

## Abstract

Actinic cheilitis (AC) is considered a potentially malignant disorder characterized by epithelial atrophy, chronic inflammation, and the progressive deterioration of the tissue architecture of the vermilion border of the lip. Currently, there is no effective minimally invasive treatment to reverse the progression of this pathology. The use of polydeoxyribonucleotide (PDRN) has demonstrated benefits across various dermatologic and wound-healing contexts due to its regenerative, angiogenic, and anti-inflammatory properties. This study aims to evaluate the healing effect of PDRN on AC lesions. This report describes the case of a 59-year-old woman with an ulcerated lesion persisting for more than two to three weeks on her lower lip, diagnosed as AC. Previous treatments with creams were unsuccessful and led to recurrence. A single subdermal infiltration of PDRN resulted in the restoration of epithelial integrity, reduced tissue fragility, and a significant improvement in oral function; that is, the patient can laugh and eat without pain after 21 days of treatment. These findings suggest that PDRN therapy is a minimally invasive option for tissue repair in AC, with potential preventive value against progression to squamous cell carcinoma. Further clinical studies are needed to confirm these results.

## Introduction

Actinic cheilitis (AC) is considered a potentially malignant disorder, or an initial and superficial form of squamous cell carcinoma (SCC) that affects the vermillion border of the lip [1]. The prevalence of AC in the general population is estimated to be between 0.3% and 3.7% [2]; however, in the sun-exposed population, such as sugarcane workers, the prevalence increases to 9.1% and has a malignant potential in 11-36% of cases [3].

The vermillion of the lips is particularly susceptible to UV radiation, especially the lower lip, due to its structural and topographic characteristics as a transitional tissue between skin and oral mucosa. For this reason, the lip vermillion comprises a stratified squamous thin epithelium with few cell layers, which contains less melanin than skin and lacks sweat and sebaceous glands, which play a protective role against UV light [4].

Reactive oxygen species (ROS) are the principal cause of UV-induced damage in the skin [5]. UV radiation can stimulate pro-inflammatory factors, such as nuclear factor-kappa B (NF-kB), and promote inflammasome formation [6]. It can induce DNA damage and mutations in keratinocytes, the most common cell type in the epidermis [6], in the early stage of a pre-cancerous lesion of SCC. At the histopathological level, an increased thickness of the keratin layer (hyperkeratosis, parakeratosis), atrophy, or thickening of the stratum spinosum are often present. Still, the basement membrane is intact [7].

Preventive treatment for AC aims to reduce the risk of malignant transformation from AC to SCC while maintaining lip function and cosmesis. In the early stage of AC, protection from UV radiation and topical therapies such as 5-fluorouracil, imiquimod, or diclofenac cream are recommended; however, there is no consistent evidence of the efficacy of these non-surgical treatments [8]. To consolidate the medical evidence on treatments for AC, a systematic review concluded that the remission rate for non-surgical treatments is only 65.9% [9].

Polydeoxyribonucleotide (PDRN) is emerging as a highly promising biomaterial in regenerative medicine and dermatology. Derived from salmon sperm, PDRN is composed of a mixture of deoxyribonucleotides that play a crucial role in cellular regeneration and tissue repair [10]. The mechanism of action of PDRN is not completely clear; however, PDRN has been associated with anti-inflammatory effects (e.g., inhibition of NF-kB and IL-6) and with the stimulation of collagen synthesis [11,12]. In vivo studies, particularly in diabetic mouse models, confirmed accelerated wound closure, improved epithelialization, increased vascularization, and modulation of inflammatory markers [13]. The effect of PDRN on a chronic non-healing wound was reported in a 28-year-old male patient refractory to negative pressure wound therapy, skin graft, or growth factors. The PDRN ameliorated wound healing by enhancing tissue repair, cell growth, and angiogenesis [14].

Considering the pathogenesis mechanisms of AC and the proposed molecular pathways of skin repair in PDRN, this article presents a case report evaluating the effect of PDRN on the healing of an early-stage AC.

## Case presentation

A 59-year-old woman, who is a fan of outdoor sports and has no family history of oral lesions, skin cancer, systemic chronic diseases, or prescribed medications, presented with an ulcerated lesion in her lower lip that had persisted for more than two to three weeks without healing, along with extremely dry lips. The patient expressed discomfort, pain, and, sometimes, bleeding when eating or laughing.

The diagnosis of AC was established by a biopsy of mucosa lined by slightly acanthotic squamous epithelium. In the histological analysis, hyperkeratosis, parakeratosis, and leucocyte infiltration in the intracorneal stratum were reported by the Pathological Anatomy Department at Clínica Los Andes, Santiago. Previous treatments for AC consisted of retinoid creams, without significant improvement in wound cicatrization.

The patient was informed about the benefits and risks of the experimental procedure before its execution and signed an informed consent form.

**Figure 1:**
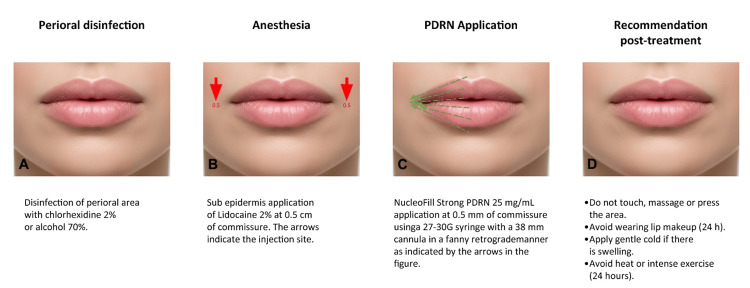
Step-by-step description of aesthetic treatment (A) Perioral disinfection. (B) Anesthesia; red arrows indicate the puncture points. (C) PDRN (polydeoxyribonucleotide) application; green arrows indicate the direction of the retrograde fanny technique with the cannula. (D) Recommendation post-treatment. Original image by the author.

Prior to treatment, the perioral area is disinfected with 2% chlorhexidine or 70% alcohol. The NucleoFill Strong® (PDRN 25 mg/mL; Promo Italia, Milan, Italy) was applied using a 27-30 G x 38 mm cannula in a single-entry point per hemilip at 5 mm from each commissure. Retrograde tracing was performed from the central zone toward the commissure (both the upper and lower lip), creating vectors in the subcutaneous plane and avoiding direct injections into the commissure [15]. The treatment consisted of one session with a follow-up at day 15. The entire procedure took 30 minutes (Figure 1).

**Figure 2:**
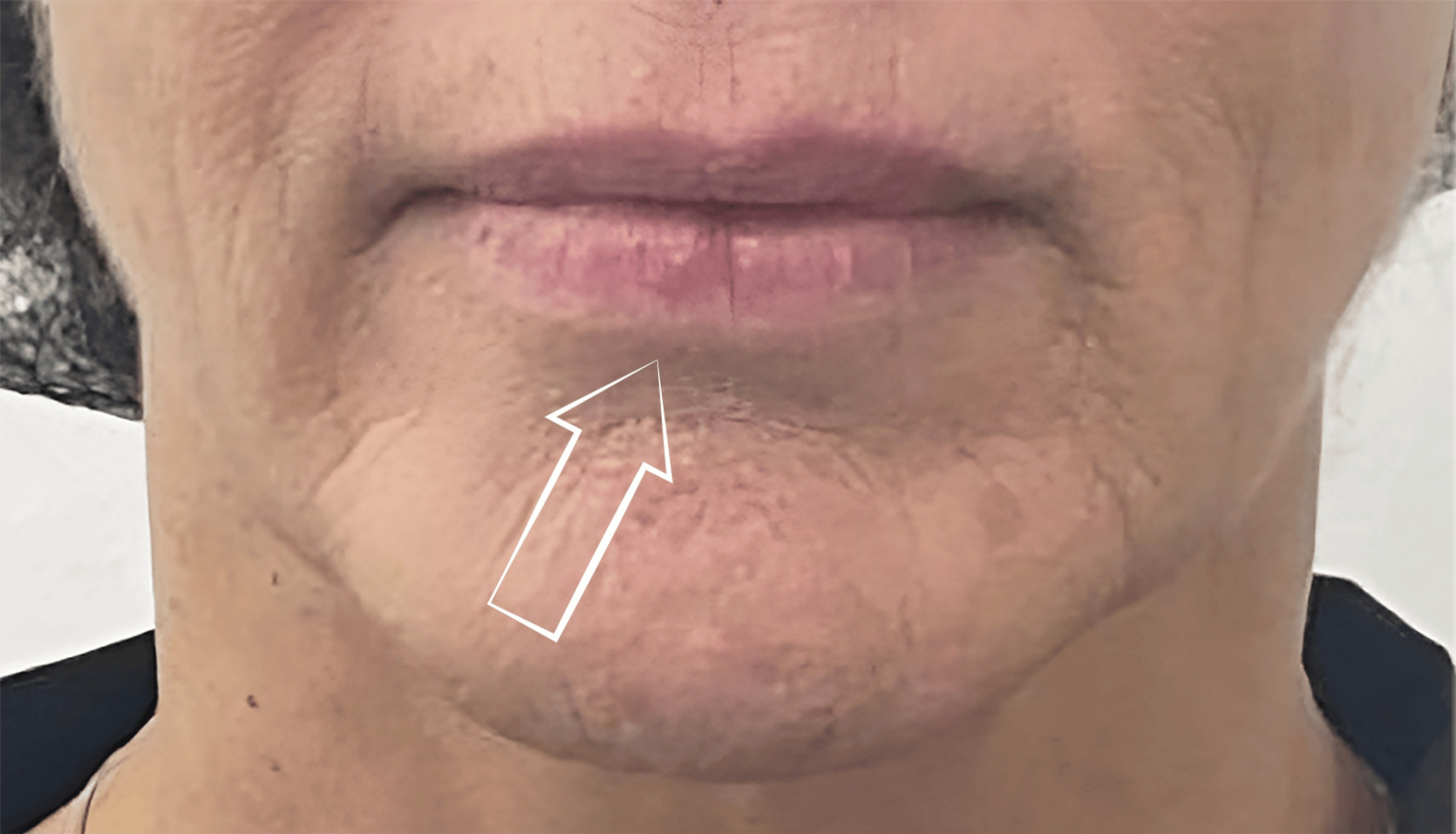
Actinic cheilitis lesion before treatment

**Figure 3:**
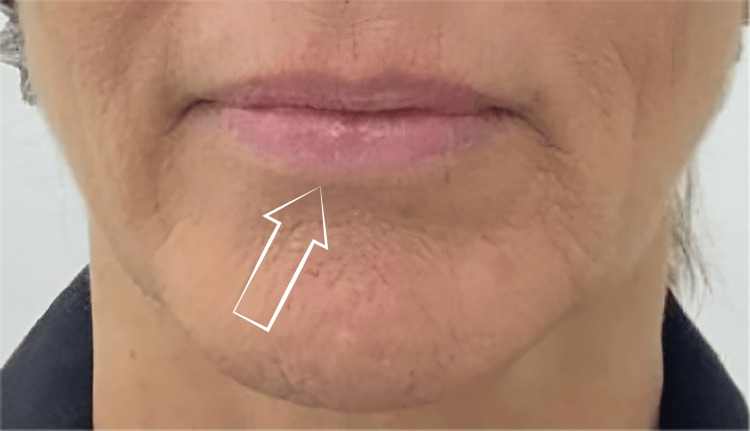
Actinic cheilitis lesion after treatment

After 15 days of treatment, the lesion size in AC decreased, and the patient reported a significant reduction in pain (Figures 2, 3). The effect of PDRN was assessed with a photonumeric lip health scale composed of three parameters: lip shine, texture, and vermilion border [16]. The baseline scores were lip shine = 3, texture = 4, and vermilion border = 3, and the post-treatment scores were lip shine = 2, texture = 2, and vermilion border = 2. After treatment, the patient reported that her lips no longer cracked or hurt as they used to. She perceived that the change is more than aesthetic; it’s a relief. She feels that she has at last recovered part of her own identity.

## Discussion

Although clinical evidence for the use of PDRN in aesthetic medicine and dermatology is incipient, its use is growing year by year, highlighting the need to standardize procedures based on scientific evidence [17]. Although the potential of PDRN as a skin repair agent has been described, evidence on its efficacy and safety has been building gradually, with studies involving small numbers of people. Recently, a case report (of five women) was published aimed at evaluating the injection of PDRN into the lips for lip rejuvenation without volume increase. All patients reported high satisfaction immediately after treatment and a marked improvement in lip texture and elasticity at six months [18].

Currently, there is an unmet need in the treatment of AC due to invasive therapies like carbon dioxide laser ablation and vermilionectomy, which were associated with the best outcomes but did not completely avoid recurrences, while minimally invasive therapies have less risk of serious adverse events, but the rate of recurrences is higher [9].

In the present case report, we observed an improvement in the AC healing lesion and in lip shine, texture, and vermilion border definition after 21 days of treatment. Our results are consistent with the findings of Rho et al., who evaluated the efficacy and safety of PDRN for lip dryness. The PDRN significantly improved both the vermilion wrinkles and roughness at all time points compared to baseline in 27 enrolled women in the clinical study. The adverse event reactions were mainly mild and transient [19].

This study has two principal limitations: the lack of follow-up beyond 21 days to assess the durability of the beneficial effect and the use of objective parameters to measure the treatment effect. Despite these constraints, this report opens a new line of investigation for minimally invasive regenerative therapies in AC.

The absence of precancerous cells (keratinocyte dysplasia) on histological examination, the recurrence of the AC lesion in our patient, and the beneficial potential of PDRN in dermatology were the main criteria in the present case report for testing PDRN therapy. The favorable results of this study encourage us to continue our research to confirm the use of PDRN as a novel treatment to reverse the progression of AC lesions.

## Conclusions

The PDRN therapy reduces pain, restores vermilion integrity, and recovers functional movements of the lips, suggesting that PDRN may offer an innovative option to restore tissue damaged by UV radiation, particularly in cases where ablative treatments are undesirable.

The findings support the hypothesis that deep biostimulation may play a preventive role by enhancing the epithelial quality of tissue considered at oncological risk. Further studies will be essential to confirm this therapeutic potential and to define standardized treatment protocols.
